# Retraction Note: Optimized extraction, composition, antioxidant and antimicrobial activities of exo and intracellular polysaccharides from submerged culture of *Cordyceps cicadae*

**DOI:** 10.1186/s12906-018-2344-0

**Published:** 2018-10-16

**Authors:** Sapan Kumar Sharma, Nandini Gautam, Narender Singh Atri

**Affiliations:** 1 Department of Plant Pathology, CSK, Himachal Pradesh Agriculture University, Palampur, 176 062 India; 2 Centre for Environment Science and Technology, School of Environmental and Earth Sciences, Central University of Punjab, Bathinda, 151 001 India; 3 Department of Botany, Punjabi University Patiala, Patiala, Punjab 147 002 India

## Retraction Note

The editor has retracted this article [1] due to overlap with a previously published article [2]. Both articles also appear to use the same image representing different fungal mycelial growths. S.K. Sharma and N.S. Atri do not agree to this retraction. N. Gautam has not responded to correspondence from the editor about this retraction.
